# Interpreting Meta-Analyses of Genome-Wide Association Studies

**DOI:** 10.1371/journal.pgen.1002555

**Published:** 2012-03-01

**Authors:** Buhm Han, Eleazar Eskin

**Affiliations:** 1 Department of Computer Science, University of California Los Angeles, Los Angeles, California, United States of America; 2 Department of Human Genetics, University of California Los Angeles, Los Angeles, California, United States of America; University of Washington, United States of America

## Abstract

Meta-analysis is an increasingly popular tool for combining multiple genome-wide association studies in a single analysis to identify associations with small effect sizes. The effect sizes between studies in a meta-analysis may differ and these differences, or heterogeneity, can be caused by many factors. If heterogeneity is observed in the results of a meta-analysis, interpreting the cause of heterogeneity is important because the correct interpretation can lead to a better understanding of the disease and a more effective design of a replication study. However, interpreting heterogeneous results is difficult. The standard approach of examining the association p-values of the studies does not effectively predict if the effect exists in each study. In this paper, we propose a framework facilitating the interpretation of the results of a meta-analysis. Our framework is based on a new statistic representing the posterior probability that the effect exists in each study, which is estimated utilizing cross-study information. Simulations and application to the real data show that our framework can effectively segregate the studies predicted to have an effect, the studies predicted to not have an effect, and the ambiguous studies that are underpowered. In addition to helping interpretation, the new framework also allows us to develop a new association testing procedure taking into account the existence of effect.

## Author Summary

Genome-wide association studies are an effective means of identifying genetic variants that are associated with diseases. Although many associated loci have been identified, those loci account for only a small fraction of the genetic contribution to the disease. The remaining contribution may be accounted by loci with very small effect sizes, so small that tens of thousands of samples are needed to identify them. Since it is costly to conduct a study collecting such a large sample, a practical alternative is to combine multiple independent studies in a single analysis called meta-analysis. However, many factors, such as genetic or environmental factors, can differ between the studies combined in a meta-analysis. These factors can cause the effect size of the causal variant to differ between the studies, a phenomenon called heterogeneity. If heterogeneity exists in the data of a meta-analysis, interpreting the meta-analysis results is an important but difficult task. In this paper, we propose a method that helps such interpretation, in addition to a new association testing procedure that is powerful when heterogeneity exists.

## Introduction

Meta-analysis is a tool for aggregating information from multiple independent studies [1]–[3]. In genome-wide association studies (GWASs) [4], the use of meta-analysis is becoming more and more popular because one can virtually collect tens of thousands of individuals that will provide power to identify associated variants with small effect sizes [5]–[7]. Several large scale meta-analyses have been performed for diseases including type 1 diabetes [8], type 2 diabetes [9]–[11], bipolar disorder [12], Crohns disease [13], and rheumatoid arthritis [14], and have identified associations not revealed in the individual studies.

In meta-analyses, the effect size between studies may differ and this difference, or heterogeneity, can be caused by many factors [15]–[18]. If the populations are different between studies, the genetic factors can cause heterogeneity [19], [20]. If the subjects are from different regions, the environmental factors can cause heterogeneity [21]. Even if the true effect size is invariant, the design factors can also cause a phenomenon that looks like heterogeneity, what is often called the statistical heterogeneity [22]. If the linkage disequilibrium structures are different between studies, the collected marker can show heterogeneity [23]. If the studies use different genotyping platforms, different imputation accuracies and different genotyping errors can cause heterogeneity [24].

In current meta-analyses of genome-wide association studies, heterogeneity is often observed in the results [9]–[11], [13], [17]. Interpreting the cause of such heterogeneity is important. If the heterogeneity is caused by either genetic or environmental factors, understanding the cause of heterogeneity can help our understanding of the disease mechanism. If the heterogeneity is statistical heterogeneity caused by the design factors, understanding the cause of heterogeneity is crucial in designing a replication study so that we can eliminate the design factors that can hinder the revelation of the true effect in the replication study.

However, interpreting heterogeneous results is difficult. One standard approach is to examine the association p-values of the studies. The inherent limitation of this approach is that a non-significant p-value is not evidence of the absence of an effect. Thus, a p-value does not provide the full information necessary for the interpretation whether or not there is an effect in the study. Another standard approach is to plot observed effect sizes and their confidence intervals of all studies [17], [25], [26]. This plot can be overly complicated when the number of studies is large and does not provide a single estimate that represents the existence of an effect in each study. The limitation of both approaches is that they use classical estimates that are calculated using only the data of each single study. That is, they utilize only within-study information.

In this paper, we propose a framework facilitating the interpretation of the results of a meta-analysis. Our framework is based on a new statistic termed the *m-value* which is the posterior probability that the effect exists in each study. Plotting the new statistic together with p-values in a two-dimensional space helps us distinguish between the studies predicted to have an effect, the studies predicted to not have an effect, and the ambiguous studies that are underpowered. We name this plot a P-M plot. In this framework, the outlier studies showing distinct characteristics from the other studies are easily identified, as we demonstrate using data from type 2 diabetes and Crohns disease meta-analyses [10], [13]. Our new statistic is fundamentally different from traditional estimates based on the data of single studies. We use all studies simultaneously to calculate the new statistic based on the assumption that the effect sizes are similar if the effect exists. Thus, we utilize cross-study information as well as within-study information.

In addition to helping interpretation, the new framework allows us to develop a new association testing procedure which takes into account the presence or absence of the effect. The new method called the binary effects model is a weighted sum of z-scores method [5] assigning a greater weight to the studies predicted to have an effect and a smaller weight to the studies predicted to not have an effect. Application to the Crohns disease data [13] shows that the new method gives more significant p-values than previous methods at certain loci already identified as associated.

The new method is available at http://genetics.cs.ucla.edu/meta.

## Methods

### Binary Effects Assumption

In our framework, we use a simplified model to describe heterogeneity among the studies which makes two assumptions. The first assumption is that *effect is either present or absent in the studies*. This assumption is different from the traditional assumption assuming normally distributed effect sizes [27]–[29]. Our assumption is inspired by the phenomenon that the effect sizes are sometimes observed to be much smaller in some studies than in the others. It is reported that different populations can cause such phenomenon [19], [20], [30], [31]. For example, the homozygosity for *APOE* 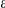
4 variant is known to confer fivefold smaller risk of Alzheimer disease in African Americans than in Asians [19], [30]. The HapK haplotype spanning the *LTA4H* gene is shown to confer threefold smaller risk of myocardial infraction in the populations of Europeans decent than in African Americans [31]. The *HNF4A* P2 promoter variants are shown to be associated with type 2 diabetes in Ashkenazi and the results have been replicated [20]. However, in the same study, the same variants did not show associations in four different cohorts of UK population suggesting a heterogeneous effect. Gene-environmental interactions can also cause such phenomenon. If a study lacks an environmental factor necessary for the interaction, the observed effect size can be much smaller in that study. It is generally agreed that the gene-environmental interactions exist in many diseases such as cardio vascular diseases [32], respiratory diseases [33], and mental disorders [34].

The second assumption is that if the effect exists, the effect sizes are similar between studies. We call these two assumptions together the *binary effects assumption*. While other types of heterogeneity structures are possible such as arbitrary effect sizes, for identifying which studies have an effect and which studies do not have an effect, we expect that this model will be appropriate.

### M-Value

We propose a statistic called the *m-value* which is the posterior probability that the effect exists in each study of a meta-analysis. Suppose that we analyze 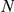 studies together in a meta-analysis. Let 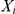 (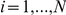) be the observed effect size of study 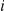 and let 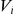 be the estimated variance of 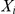. It is a common practice to consider 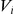 the true variance. In the current GWASs, the distribution of 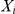 is well approximated by a normal distribution due to the large sample sizes. Let 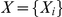 denote the observed data.

If there is no effect in study 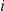,
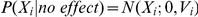
where 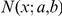 is the probability density function of a normal distribution whose mean is 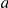 and the variance is 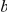. If there is effect in study 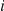,
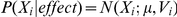
where 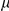 is the unknown true effect size.

Since we want a posterior probability, the Bayesian framework is a good fit. We assume that the prior for the effect size is
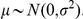
A possible choice for 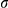 in GWASs is 0.2 for small effect and 0.4 for large effect [35], [36].

Let 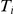 be a random variable which has a value 1 if study 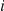 has an effect and a value 0 if study 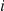 does not have an effect. Let 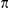 be the prior probability that each study will have an effect such that
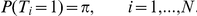
Then we assume a beta prior on 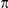


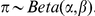
Through this paper, we use the uniform distribution prior (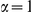 and 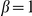), but other priors can also be chosen.

Let 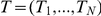 be the vector indicating the existence of effect in all studies. 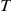 can have 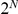 different values. Let 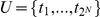 be the set of those values.

Our goal is to estimate the m-value 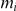, the posterior probability that the effect exists in study 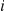. By the Bayes' theorem,
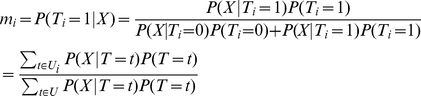
(1)where 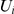 is a subset of 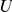 whose elements' 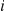
th value is 1. Thus, we only need to know for each 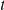 the posterior probability of 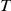,
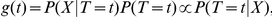
consisting of the probability of 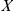 given 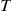 and the prior probability of 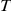.

The prior probability of 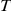 is
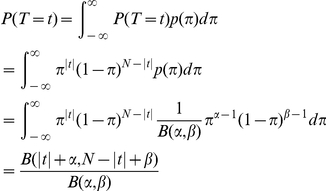
where 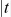 is the number of 1's in 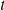 and 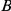 is the beta function.

And the probability of 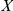 given 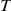 is
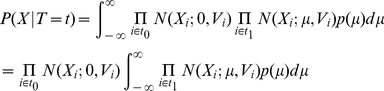
(2)where 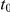 is the indices of 0 in 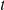 and 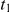 is the indices of 1 in 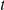. We can analytically work on the integration to obtain
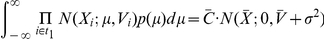
where
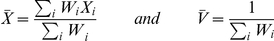
where 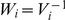 is the inverse variance or precision. The summations are all with respect to 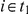.


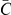
 is a scaling factor such that
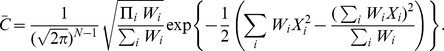
The details of the derivation is in Text S1 in Supporting Information S1. As a result, we can calculate 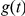 for every 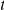 and therefore obtain 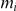 for each study 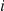.

#### MCMC

The drawback of the exact calculation of m-value is that we need to iterate over all 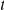 which is exponential to 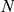. This is not problematic in most of the current meta-analyses of GWASs, but will be problematic in future studies if 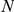 increases over several tens. Therefore, here we propose a simple Markov Chain Monte Carlo (MCMC) method to estimate m-value.

We propose the following Metropolis-Hastings algorithm [37].

1. Start from a random 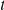.
2. Choose a next 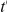.
3. If 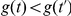, move to 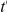. Otherwise, move to 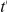 with probability 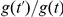.
4. Repeat from step 2.

The set of moves we use for choosing 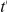 is 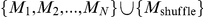. 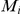 is a simple flipping move of 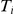 between 0 and 1. 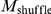 is a move that shuffles the values of 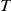. This move is introduced to avoid being stuck on one mode in a special case that there are two modes which can happen when the observed direction of the effect is opposite in some studies. At each step, we randomly choose a move from this set assuming a uniform distribution. We allow 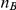 burn-in and sample 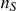 times. After sampling, 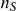 samples gives us an approximation of the distribution over 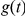, which subsequently gives the approximations of m-values by the formula (1).

#### Interpretations and predictions

The m-value has a valid probabilistic interpretation that it is the posterior probability that the effect exists in each study under our binary effects model. If we are to choose studies predicted to have an effect and studies predicted to not have an effect, a threshold is needed. In this paper, we use the threshold of m-value 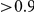 for the former and m-value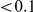 for the latter. Although this thresholding is arbitrary, the actual level of threshold is often not of importance because outlier studies showing different characteristics from the other studies usually stand out in the plotting framework described below.

#### Relationship to PPA

The m-value is closely related to the posterior probability of association (PPA) based on the Bayes factor (BF) [35] in the sense that the presence and absence of effects are essentially describing the same things as the alternative and null models in the association testing. There are two fundamental differences. First, in the usual PPA, the prior probability of association (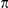) is given by a point prior which is usually a very small value in GWAS reflecting the fact that the true associations are few. In our framework, we focus on interpreting meta-analysis results after we find associations using meta-analysis. Thus, 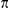 reflects our belief on the effect *conditioned on* that the associations are already significant. For this reason, we need not use a very small value but instead choose to use a distribution prior. Second, the PPA is calculated for each study separately. However, the m-value is calculated using all studies simultaneously utilizing cross-study information. Thus, if the binary effects assumption approximates the truth, the m-value is more effective in predicting effects than the PPA or equivalently the BF, as we show by simulations in Results.

### P-M Plot

**Figure 1:**
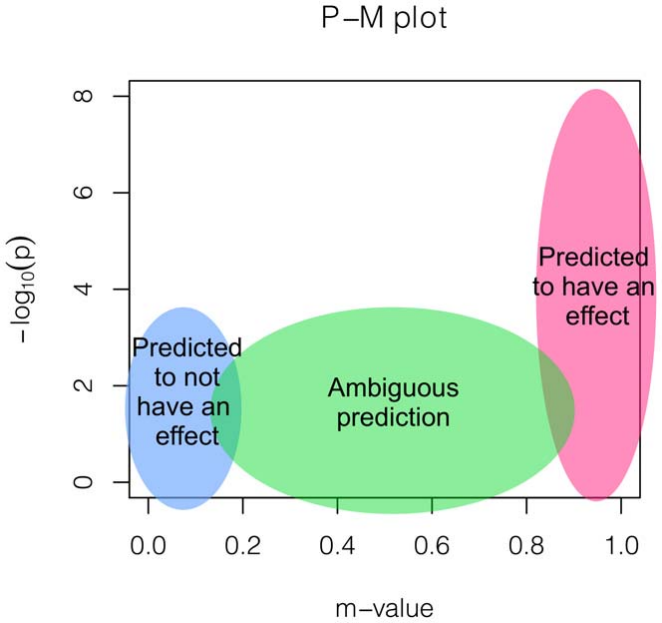
A figure depicting the interpretations based on a P-M plot.

We propose plotting the studies' p-values and m-values together in two dimensions. This plot, which we call the P-M plot, can help interpreting the results of a meta-analysis. Figure 1 shows that how to interpret such a plot. The right-most (pink) region is where the studies are predicted to have an effect. Often, a study can be in this region even if the p-value is not very significant. The left-most (light-blue) region is where the studies are predicted to not have an effect. This suggests that the sample size is large but the observed effect size is close to zero, suggesting a possibility that there exists no effect in that study. The middle (green) region is where the prediction is ambiguous. A study can be in this region because the study is underpowered due to a small sample size. If the sample size increases, the study will be drawn to either the left or the right side.

If the binary effects assumption does not hold, a study can sit in an unexpected region and a careful interpretation is necessary. For example, if the effects are significant but the effect sizes are in opposite direction in some studies, the studies can sit in the unusual top left region. However, such case will be rare and may be a result of the strand errors.

### Binary Effects Model

We propose a new type of random effects model meta-analysis approach called the binary effects model. If the binary effects assumption holds, that is, if the effect is either present or absent in the studies, taking into account this pattern of heterogeneity in the association testing procedure can increase power compared to the general RE approach [23]. The binary effects model we propose is the weighted sum of z-scores method [5] where the m-values are incorporated into the weights. Intuitively, this is equivalent to assigning a greater weight to the studies predicted to have an effect and a smaller weight to the studies predicted to not have an effect.

Let 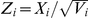 be the z-score of study 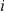. The common form of the weighted sum of z-scores statistic for the fixed effects model is
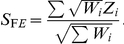
In many cases, the weight 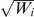 approximates to the form 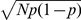 where 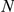 is the sample size and 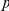 is the minor allele frequency [23]. When the minor allele frequency is similar between studies, the weight 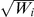 approximates to the popular form of 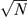 [5].

The binary effects model statistic we propose is
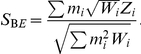
Our method is an empirical approach that uses 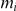 estimated from the data as the prior weight for each study. Since the m-value is estimated using all studies, our approach can be thought of as gathering information from all studies and distributing back to each study in the form of weight. We choose this approach because of its simple formulation.

Since 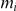 is not independent of 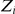, the statistic does not follow a normal distribution. Thus, the p-value is obtained using sampling which can be inefficient. We use two ideas to expedite the sampling. First, we propose an importance sampling procedure which is more efficient than the standard sampling. Second, we use an efficient approximation of m-value. See Text S2 and S3 in Supporting Information S1 for details.

### Simulation Framework

In order to evaluate our methods, we use the following simulation approach. Assuming a minor allele frequency, a relative risk, and the number of individuals of 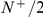 cases and 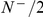 controls, a straightforward simulation approach is to sample 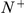 alleles for cases and 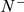 alleles for controls according to the expected minor allele frequencies in the cases and controls respectively [38]. However, since we perform extensive simulations assuming thousands of individuals, we use an approximation approach that samples the minor allele count from a normal distribution and rounds it to the nearest non-negative integer.

### Web Resources

The URL for methods presented herein is as follows:

http://genetics.cs.ucla.edu/meta

## Results

### Simulation of M-Values

We demonstrate a simple simulation example showing how m-value behaves depending on the presence and absence of the effect and the sample size. First, we make the following assumptions throughout all of the experiments in this paper. We assume that the minor allele frequency of the collected marker is 0.3. We assume that the equal number of cases and controls are collected and refers to the total number of individuals as sample size 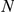. We also assume a very small disease prevalence when we calculate the expected minor allele frequencies for cases and controls given a relative risk 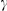. For the details how the expected values are calculated, see Han and Eskin [23]. Note that these assumptions are not critical factors affecting our simulation results. In all experiments, the random effects model (RE) denotes the RE method of Han and Eskin [23]. We omit the results of the conventional RE method [15] because they are highly conservative [23]. Throughout this paper, we use the following priors for calculating m-values. We use 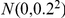 for the prior of the effect size (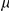). We use the uniform distribution prior, 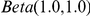, for the prior of the existence of effect (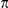).

In this simulation example, we assume four different types of studies. The first type is a large study having an effect (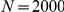 and 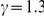). The second type is a small study having an effect (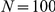 and 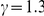). The third type is a large study not having an effect (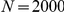 and 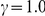). The fourth type is a small study not having an effect (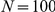 and 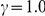). We generate two studies per each type, constructing a simulated meta-analysis set of total eight studies. We accept this simulation set only if none of eight studies' p-values exceeds the genome-wide threshold (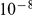) but the meta-analysis p-value calculated by the RE approach exceeds the genome-wide threshold. Otherwise, we repeat. We construct 1,000 meta-analysis sets.

**Figure 2:**
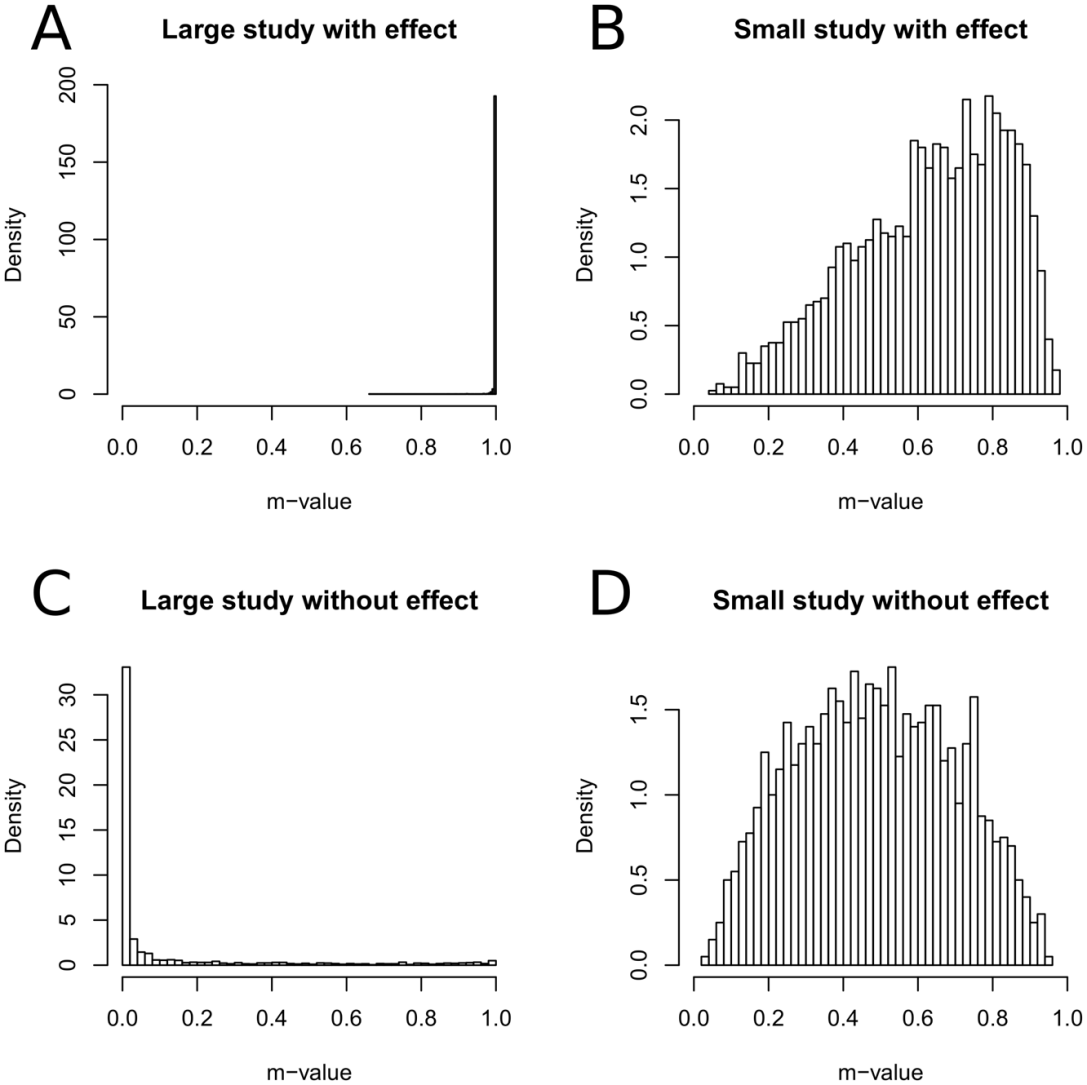
Histograms of m-values of different types of studies. We assume four types of studies which is the all four combinations of large sample (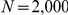) and small sample (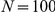), and effect (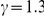) and no effect (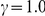). We repeatedly simulate a meta-analysis of eight studies, two studies per each type, and calculate the m-values of the studies.

Given this simulated data, we plot the histogram of m-values for each type of studies separately in Figure 2. Figure 2A shows that almost all (99.9%) of large studies with an effect are concentrated on large m-values (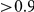), showing that the m-values effectively predict that the effect exists in the studies. Figure 2C shows that a large amount (78.6%) of large studies without an effect are concentrated on small m-values (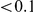). Figure 2B and 2D show that when the sample size is small, m-value tends to the mid-range regardless of the effect, suggesting that the studies are underpowered to determine the presence of an effect.

### Comparison of P-Value, M-Value, and BF

In this experiment, we compare the p-value, m-value, and BF by measuring how well they predict which studies have an effect and which studies do not have an effect. We assume a meta-analysis of 10 studies where the effect is either present (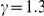) or not. We randomly pick the number of studies having an effect (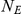) from a uniform distribution ranging from 1 to 9, and randomly decide which studies have an effect. We randomly pick the sample size of each study from a uniform distribution between 500 and 2,000. Given the sample sizes and the effect sizes, we generate a meta-analysis study set. We accept the meta-analysis set only if none of the studies' p-values exceeds the genome-wide threshold (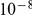) and the meta-analysis p-value exceeds the genome-wide threshold. We repeat until we construct 1,000 meta-analysis sets.

We examine each of 10,000 studies included in the simulated 1,000 meta-analysis sets. For each study, we calculate the p-value, m-value, and BF. We use the asymptotic BF of Wakefield [39] assuming the same prior distribution 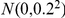 about the effect size as the m-value. Then we evaluate the performance of each statistic as follows. To evaluate the performance of m-value, we fix an arbitrary threshold 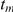 so that we predict the studies having m-value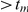 to have an effect. Since we know the underlying truth if the effect exists or not in each study, we can measure what proportion of the studies actually having an effect is correctly predicted to have an effect (true prediction rate) and what proportion of the studies actually not having an effect is incorrectly predicted to have an effect (false prediction rate). Then we change the threshold 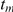 to draw a curve between the true prediction rate and the false prediction rate, which is often called the receiver-operating-characteristic (ROC) curve. We do the same analysis for p-value and BF.

**Figure 3:**
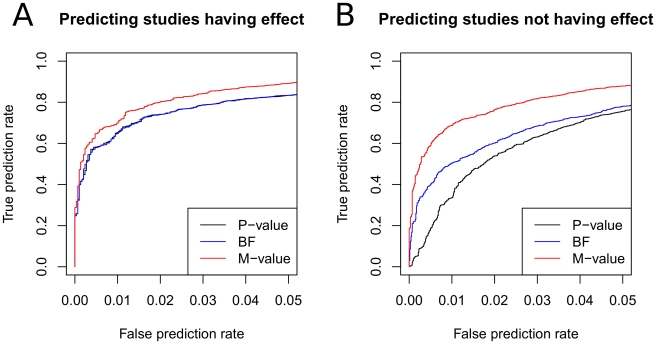
Comparison of prediction accuracies of p-value, m-value, and BF. We simulate 1,000 meta-analysis of 10 studies with varying sample sizes where only a subset of the studies have an effect. Given 10,000 studies, we threshold each statistic to predict the studies having an effect and the studies not having an effect, and vary the threshold to draw the ROC curves. In A, true prediction rate is the proportion of the studies actually having an effect that are correctly predicted to have an effect and false prediction rate is the proportion of the studies actually not having an effect that are incorrectly predicted to have an effect. In B, true and false prediction rates are similarly defined but in the direction of predicting studies not having an effect. For BF, we use the asymptotic BF of Wakefield [39] with prior 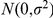 where 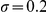.

Figure 3A shows that m-value is superior to p-value and BF in predicting the studies having an effect. This is because m-value can utilize the cross-study information when the binary effects assumption holds. The performances of p-value and BF are almost identical.

Next, we evaluate the performance of the statistics in predicting studies not having an effect. The experiment is exactly the same as the previous experiment except that, given a threshold 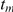, we predict the studies having m-value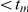 to not have an effect. We similarly draw the ROC curves for the three statistics. True and false prediction rates are defined similarly for the objective of predicting the studies not having an effect.

Figure 3B shows that the m-value is even more superior to the other statistics in this experiment than in the previous experiment. The p-value shows the most inferior performance. This is expected because p-value is designed for detecting the presence of an effect but not for detecting the absence of an effect. That is, a non-significant p-value is not evidence of the absence of an effect but can be the result of a small sample size. On the other hand, the BF testing for the absence of an effect is just the reciprocal of the BF testing for the presence of an effect. Thus, the same BF can be used for both purposes. Although the BF performs better than the p-value, the m-value is even more superior. The relative performance gain of the m-value compared to the BF is due to the cross-study information utilized.

### P-M Plot: Type 2 Diabetes Data

We apply our P-M plot framework to the real data of the meta-analysis of type 2 Diabetes (T2D) of Scott *et al.* [10]. The meta-analysis consists of three different GWAS investigations, the Finland-United States Investigation on NIDDM Genetics (FUSION) [10], the Diabetes Genetics Initiative (DGI) [11], and the WTCCC [9], [40].

In their analysis, two SNPs are shown to have a heterogeneous effect, rs8050136 and rs9300039. Ioannidis *et al.* [17] provide an insightful explanation about the heterogeneity at rs8050136. The WTCCC/UKT2D groups identified evidence for T2D and body mass index (BMI) associations with a set of SNPs including rs8050136 in the *FTO* region [40]. On the other hand, in the DGI study, the SNP rs8050136 was not significant. The explanation that Ioannidis *et al.* suggest is that the observed association at rs8050136 (*FTO*) may be mediated by its association with obesity. In fact, DGI is the only study where the BMI is matched between cases and controls, and the T2D association appears to be mediated through a primary effect on adiposity [11]. Thus, although the truth is unknown, the explanation of Ioannidis *et al.* is reasonable. Compared to rs8050136, the cause of heterogeneity at rs9300039 is less understood. It is suggested that the heterogeneity might reflect the different tag polymorphisms used in the studies [17].

**Figure 4:**
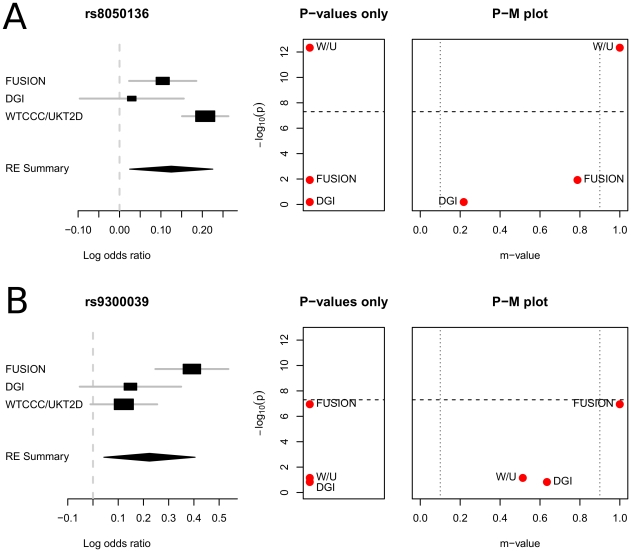
P-M plots of the type 2 diabetes meta-analysis results of Scott *et al.* [**10**]. Two associated loci showing high heterogeneity are plotted. The dashed horizontal line shows the genome-wide significance threshold. The dotted vertical lines show the prediction regions based on m-value.

To gain insights on these studies, we apply our P-M plot. Figure 4A shows the forest plot, the plot showing only the p-values, and the P-M plot for rs8050136. In the P-M plot, DGI appears to be well separated from the other two studies, even though its m-value (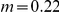) is not below the threshold (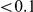). Thus, the P-M plot visualizes that DGI can have a different characteristic from the others. Such a separation is not clear in the plot showing only the p-values. In the plot showing only the p-values, DGI is close to FUSION since FUSION is also not very significant (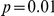). However, the m-value of FUSION is much greater (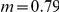) than that of DGI. This suggests that the effect is much more likely to exist in the FUSION study than in the DGI study.

Figure 4B shows the plots for rs9300039. The P-M plot shows a different pattern from the P-M plot of rs8050136. In this P-M plot, every study has an m-value greater than 0.5. Thus, no study shows evidence of no effect. Comparing the plots of rs8050136 and rs9300039 gives an interesting observation. In the plot showing only the p-values, both SNPs show a specific pattern of p-values that a single study is considerably more significant than the other two. However, despite of this similarity in the pattern of p-values, the two SNPs' P-M plots look different enough that can lead us to different interpretations. This shows that our P-M plot can provide information that is not apparent in the analysis of only the p-values.

### P-M Plot: Crohns Disease Data

We apply our plotting framework to the data of the recent meta-analysis of Crohns disease of Franke *et al.* [13]. This meta-analysis consists of six different GWAS comprising 6,333 cases and 15,056 controls, and even more samples in the replication stage. In this study, 39 associated loci are newly identified increasing the number of associated loci to 71. We apply our framework to six loci where a high level of heterogeneity is observed. Han and Eskin [23] showed that at these six loci, RE gave more significant p-values than the fixed effects model (FE).

**Figure 5:**
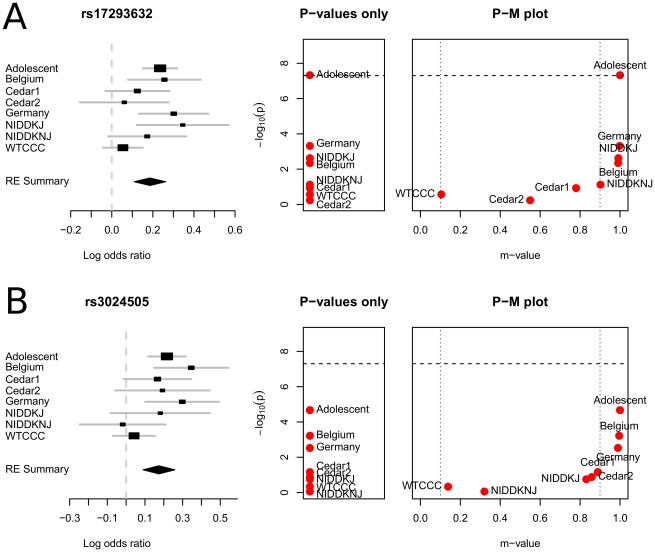
P-M plots of the Crohns disease meta-analysis results of Franke *et al.* [**13**]. Two of six associated loci showing high heterogeneity are plotted. See Figure S1 for the plots of all six loci. The names of the studies follow Franke *et al.* [13]. The dashed horizontal line shows the genome-wide significance threshold. The dotted vertical lines show the prediction regions based on m-value.

Figure 5 shows the P-M plots of two loci. See Figure S1 for the plots of all six loci. The names of the studies follow the names used in Franke *et al.* [13]. At these two loci, rs3024505 and rs17293632, the m-value of WTCCC is close to the threshold for predicting no effect. A possible explanation is that the different marker sets could have caused the statistical heterogeneity at these loci. WTCCC [40] used the Affymetrix platform while others used the Illumina platform. Although we do not further investigate this hypothesis, it is true that the P-M plots visualize an interesting outlier behavior of WTCCC at these loci. Such an observation is not clear in both the forest plot and the plot showing only p-values. In the plot showing only p-values, studies having non-significant p-values are all clustered and WTCCC is only one of them. In the forest plot, WTCCC is not the only study showing a small effect size at both loci. For example, at rs3024505, NIDDKNJ shows a smaller effect size than WTCCC. However, the m-value of WTCCC is much smaller than NIDDKNJ's because of the large sample size. Such an interaction between the sample size and the prediction can also be inferred from the forest plot since the forest plot includes the confidence interval. However, it is difficult to numerically quantify the effect of sample size on the prediction by visually examining the forest plot.

### Binary Effects Model: False Positive Rate

We estimate the false positive rate of the new binary effects model (BE). Assuming the null hypothesis of no association, we construct 5 studies of sample size 1,000 to build a meta-analysis set. We calculate the meta-analysis p-value of BE using our importance sampling procedure with 10,000 samples. We also calculate the meta-analysis p-values of FE and RE. We build 100 million sets of meta-analysis and estimate the false positive rate as the proportion of the simulated sets whose p-value exceeds a threshold. We vary the threshold levels from 0.05 to 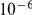. Table 1 shows that all methods including BE control the false positive rates accurately, at all threshold levels examined. When we increase the number of studies from 5 to 10, the results are essentially the same and the false positive rates are controlled (Data not shown).

**Table 1:** False positive rate of FE, RE, and BE at increasing significance thresholds.

Threshold	FE	RE	BE
5.0E-02	4.98E-02 (1.00)	4.98E-02 (1.00)	4.98E-02 (1.00)
1.0E-02	9.95E-03 (0.99)	9.92E-03 (0.99)	9.93E-03 (0.99)
1.0E-03	9.93E-04 (0.99)	9.93E-04 (0.99)	9.92E-04 (0.99)
1.0E-04	9.85E-05 (0.99)	9.87E-05 (0.99)	1.00E-04 (1.00)
1.0E-05	9.68E-06 (0.97)	9.51E-06 (0.95)	9.17E-06 (0.92)
1.0E-06	9.70E-07 (0.97)	1.04E-06 (1.04)	1.01E-06 (1.01)

The values in the parentheses are the ratio between the false positive rate and the threshold. The estimates are obtained from 100 million null panels assuming 5 studies of an equal sample size 1,000.

### Binary Effects Model: Power

We compare the power of BE to the powers of FE and RE. Assuming a meta-analysis of five studies of an equal sample size 1,000, we construct 10,000 meta-analysis sets. The power of each method is estimated as the proportion of the meta-analysis sets whose meta-analysis p-value calculated by each method exceeds the genome-wide threshold (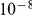).

We measure power in two different situations. First, we assume a situation that the effect is either present or absent. We decrease the number of studies having an effect (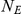) from 5 to 2. We increase the relative risk as 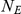 decreases, using 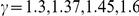 for 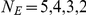 respectively, in order to show the relative performance between methods.

**Figure 6:**
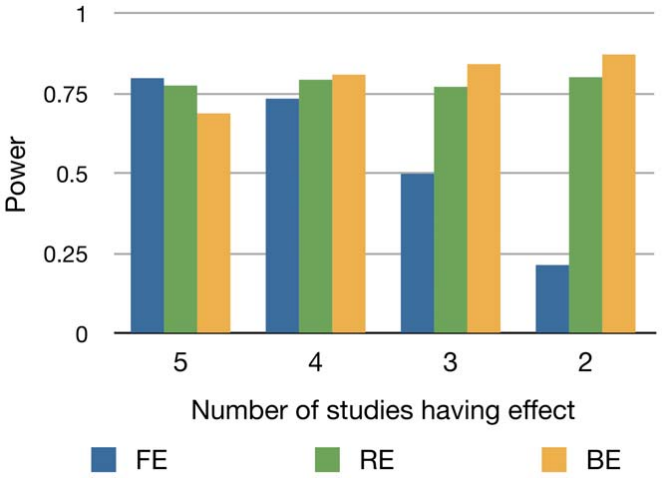
Power of FE, RE, and BE method when the number of studies having an effect varies. We assume 5 studies and gradually decrease the number of studies having an effect from 5 to 2. We assume an equal sample size of 1,000. We increase the odds ratio as the number of studies decreases to show the relative performance between methods. The power is estimated as the proportion of the simulated 10,000 meta-analysis sets whose meta-analysis p-value calculated by each method exceeds the genome-wide threshold (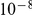).

Figure 6 shows that except for the case that there is no heterogeneity (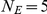), BE is the most powerful among all methods. BE is more powerful than RE, even though both are a random effects model, possibly because it learns the fact that some studies do not have an effect from the data. When there is no heterogeneity (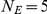), FE achieves the highest power and BE achieves the lowest power.

**Figure 7:**
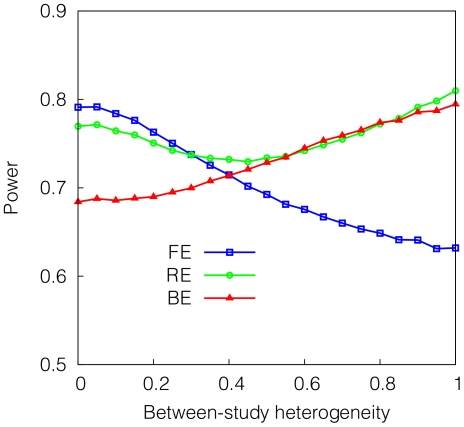
Power of FE, RE, and BE method when the effect size varies between studies in the pattern following a normal distribution. The 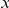-axis denotes heterogeneity 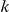 where we simulate the standard deviation of the effect size (log of relative risk) to be 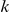 times the effect size. We assume the mean relative risk of 1.3 and assume 5 studies of an equal sample size 1,000. The power is estimated as the proportion of the simulated 10,000 meta-analysis sets whose meta-analysis p-value calculated by each method exceeds the genome-wide threshold (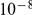).

Second, we assume a classical setting where the effect sizes follow a normal distribution. Assuming that the mean effect size of 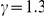, we sample the log of effect size of each study from a normal distribution having the mean 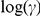 and the standard deviation 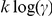 where 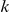 is the parameter we vary. As 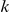 increases, the heterogeneity increases. We measure the power of each method varying 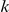 from zero to one. Figure 7 shows that in this situation, BE is generally less powerful than RE. The power difference between BE and RE is the greatest when the heterogeneity is small. As the heterogeneity increases, BE shows a similar power to RE.

### Binary Effects Model: Real Data

We apply BE to the real data of Crohns disease of Franke *et al.* [13]. Han and Eskin [23] showed that out of 69 associated loci analyzed, RE gave more significant p-values than FE at six loci where high level of heterogeneity is observed. We calculate the p-values at these loci using BE and compare to the p-values of FE and RE.

Table 2 shows that at all six loci where RE gave more significant p-values than FE, BE gives even more significant p-values. The reason why BE gives more significant p-values can be explained by examining the P-M plots of these loci in Figure 5 and Figure S1. The P-M plots show that at these loci, some studies show high m-values and some studies show low m-values, suggesting a bimodal distribution of effect size. Thus, the situation is very similar to the case that the effect is either present or not, in which case BE achieves higher power than RE as shown in Figure 6.

**Table 2:** Application of FE, RE, and BE to the Crohns disease meta-analysis results of Franke *et al.* [13].

SNP	Chr.	Position	FE p-value	RE p-value	BE p-value	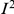
rs4656940	1	159,096,892	1.05E-06	6.91E-07	**3.99E-07**	57.01
rs3024505	1	205,006,527	7.03E-09	5.49E-09	**2.73E-09**	46.49
rs780093	2	27,596,107	1.12E-04	2.78E-05	**6.06E-06**	61.85
rs17309827	6	3,378,317	5.62E-06	4.98E-06	**3.12E-06**	22.98
rs17293632	15	65,229,650	6.17E-13	3.41E-13	**2.48E-13**	52.11
rs151181	16	28,398,018	3.32E-10	3.08E-10	**1.95E-10**	35.22

The boldface denotes the top p-value among the three methods. Only six associated loci are presented that were shown to have more significant RE p-values than FE p-values [23].

### Binary Effects Model: Accuracy of Importance Sampling

We measure how accurately the importance sampling procedure of BE estimates the p-value depending on the number of samples used. We calculate the BE p-value for the same dataset in 100 different runs to estimate the variance of the p-value estimate. Our criterion of interest is the ratio between the standard deviation of our estimate and the target p-value. For this, we use the 69 associated loci in the Crohns disease data of Franke *et al.* [13] that were previously analyzed in Han and Eskin [23]. We measure the ratio for each locus and average over all loci. We do this varying the number of samples from 1,000 to 1,000,000.

Table 3 shows that as the number of samples used for importance sampling increases, the accuracy increases. The pattern of accuracy increase is what we would usually expect in a sampling procedure; standard deviation is decreased approximately by the square root of the sample size increase. When the number of samples is 1,000, the ratio is roughly 0.5. A ratio of 0.5 is large, but can be enough for initial screening if we would apply an adaptive sampling that samples larger number of samples only for loci that are at least moderately significant (e.g. 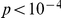).

**Table 3:** Accuracy of importance sampling depending on the number of samples.

# Samples	Stdev./P-value
1,000	0.485
10,000	0.172
100,000	0.057
1,000,000	0.018

For each given number of samples used for the importance sampling, we measure the variance of the p-value estimate of BE by running the importance sampling 100 different times for the same dataset. We use the 69 associated loci from the Crohns disease data of Franke *et al.* [13]. The ratio between the standard deviation of the estimate and the target p-value is reported, which is averaged over 69 loci.

### Binary Effects Model: Computational Efficiency

We measure the computational efficiency of the importance sampling procedure of BE. In our software, we implemented an adaptive sampling procedure that samples smaller number first (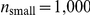) and then larger number (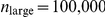) for the loci that are at least moderately significant. In the machine equipped with Intel Xeon 1.68 GHz CPU, when we use 1,000 samples in the importance sampling, calculating BE p-values of 1,000 loci for the meta-analysis of 10 studies takes 100 seconds. Thus, to calculate BE p-values of one million loci assuming that 1,000 loci among them are moderately significant, it will take approximately 30 hours which is a feasible amount of time. If the number of samples is increased to achieve better accuracy, such as 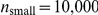 and 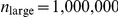, the procedure will still be efficient if one uses multiple computers or a cluster since the procedure is parallelizable.

## Discussion

We introduce a framework facilitating the interpretation of meta-analysis results based on a new statistic representing the posterior probability that the effect exists in each study. Our framework utilizes cross-study information and is shown to help interpretations in the simulations and the real data. The new statistic also allows us to develop a new association testing procedure called the binary effects model.

In the current meta-analyses of genome-wide association studies, heterogeneity is often observed and our framework will be a useful tool for interpreting such results. We expect that our framework will be even more useful in the future meta-analyses. As the number of studies in a meta-analysis grows, the chance of heterogeneity will increase [6]. Also, a meta-analytic approach can often be applied to a broader area such as to multiple diseases with similar etiology, in which case the heterogeneity is more likely to occur. Moreover, the majority of the current meta-analyses only use the fixed effects model (FE). The use of a random effects model (RE) approach [23] such as the binary effects model presented herein will increase the number of identified associations showing heterogeneity, since an RE approach is more powerful than FE for detecting associations with heterogeneity.

One limitation of our approach is that although the new statistic can predict the studies having an effect and the studies not having an effect, it does not distinguish the true heterogeneity and the statistical heterogeneity [22]. Discriminating between the two can be very difficult based on the observed data and might often be possible only by external data such as the replication studies. In that sense, our method can help discriminating them because one can come up with a hypothesis based on m-values that the heterogeneity is caused by specific design factors and then control the factors in the replication stage. The heterogeneity will disappear in the replication stage if it was due to the design factors.

Similarly to other Bayesian approaches [35], [36], the prior choice in our method can have a non-negligible effect on the predictions. For the prior of the effect size 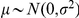, it is important to set a reasonable value 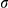 based on the prior information about the effect size. See Stephens and Balding [35] for the general guideline for this choice. For the prior of the probability that the effect exists 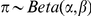, we used the uniform distribution (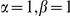) in this paper. However, different priors can also be used for different situations. If one expects that most of the studies have an effect, an asymmetric prior such as 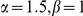 can be used. If one is certain that the studies having an effect and the studies not having an effect are mixed, a bell-shape prior such as 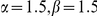 can be used. See Figure S2 for the plots of the possible choices of priors.

## Supporting Information

Figure S1 P-M plots of the Crohns disease meta-analysis results of Franke *et al.* [13]. Six loci showing high heterogeneity are plotted. The names of the studies follow Franke *et al.* [13]. The dashed horizontal line shows the genome-wide significance threshold. The dotted vertical lines show the prediction regions based on m-value.
(PDF)

Figure S2Possible choices for the prior of the probability that the effect exists. We show the uniform distribution prior (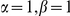), an asymmetric prior preferring the situation that all studies have an effect (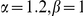), an asymmetric prior preferring the same situation even stronger (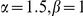), and a bell-shape prior preferring the situation that the studies having an effect and the studies not having an effect are mixed (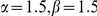).(PDF)

Supporting Information S1 This text file includes supporting information for three subjects: (Text S1) Details of the analytical calculation of m-value, (Text S2) P-value estimation using importance sampling for binary effects model, and (Text S3) Efficient m-value approximation for binary effects model.
(PDF)
