# Supplementary figures and images for: Interpreting Meta-Analyses of Genome-Wide Association Studies
Source: PLoS Genet. 2012 Mar 1;8(3):e1002555. doi: 10.1371/journal.pgen.1002555 (PMC3291559; doi:10.1371/journal.pgen.1002555)

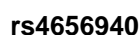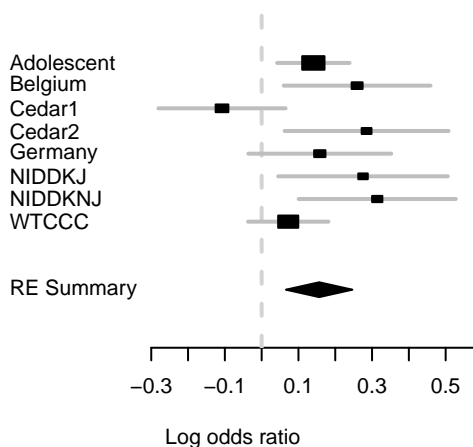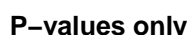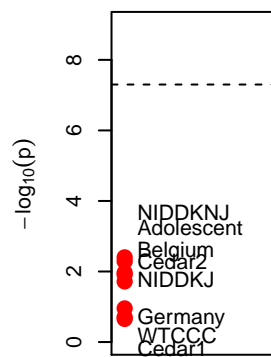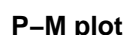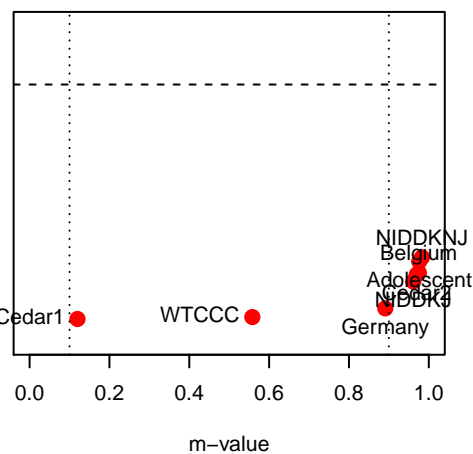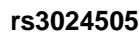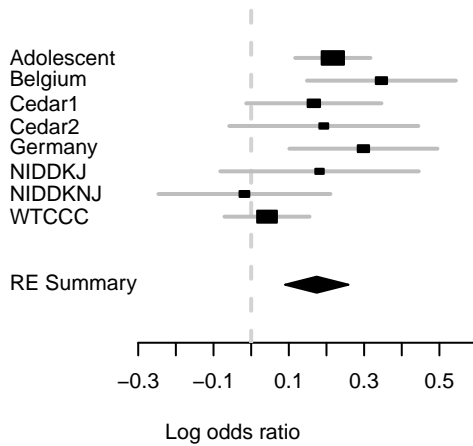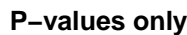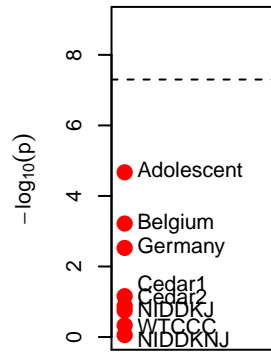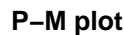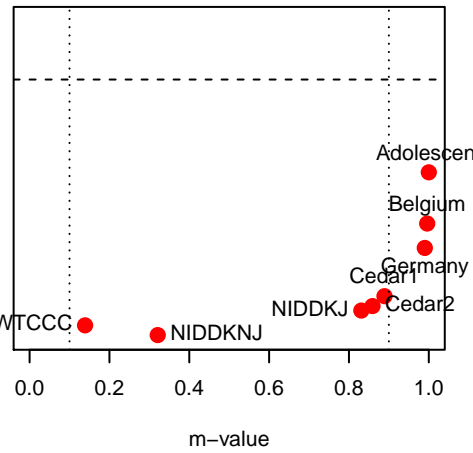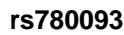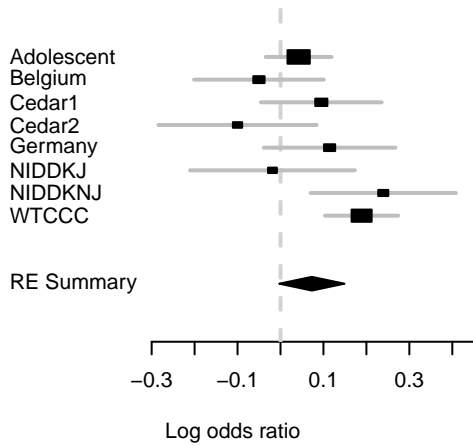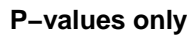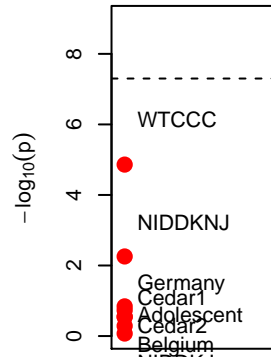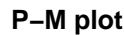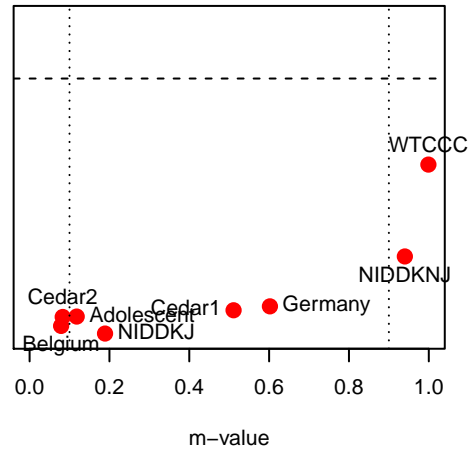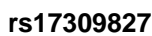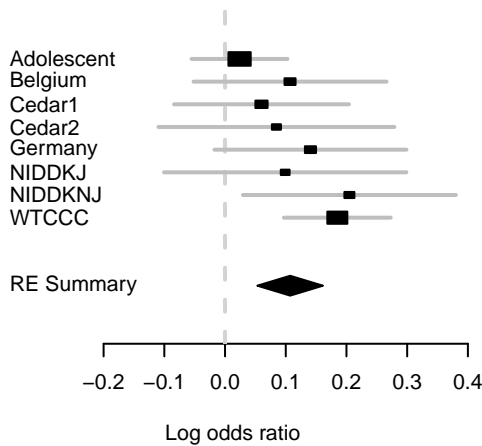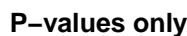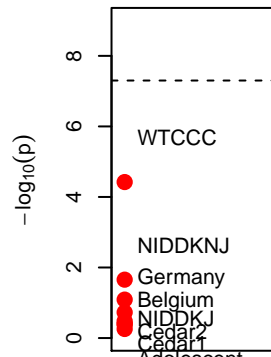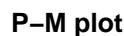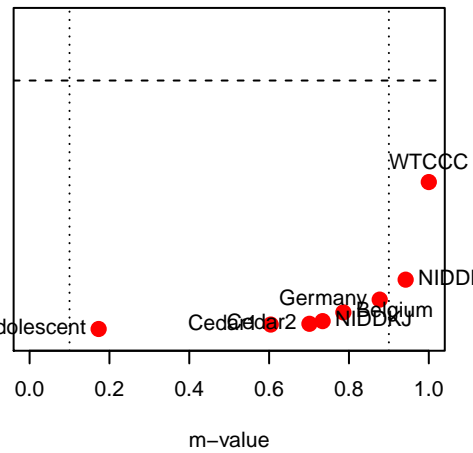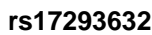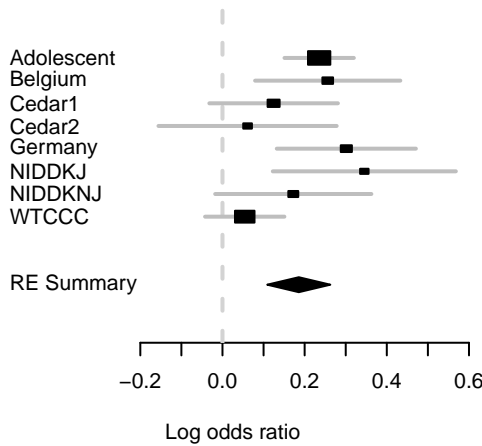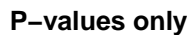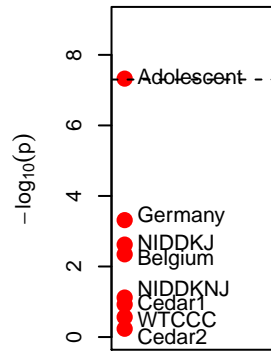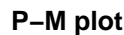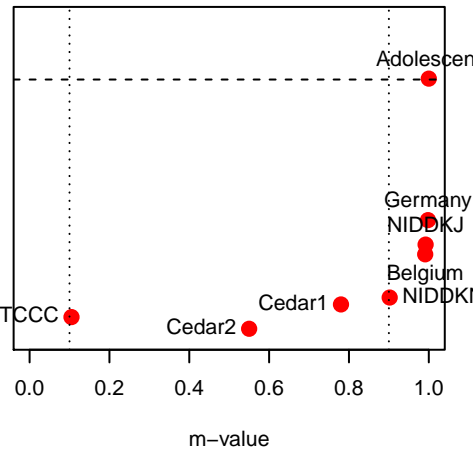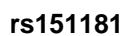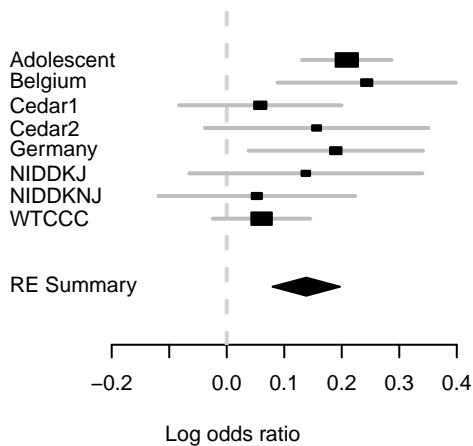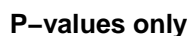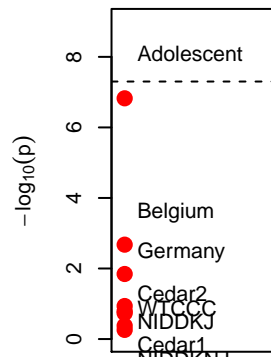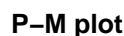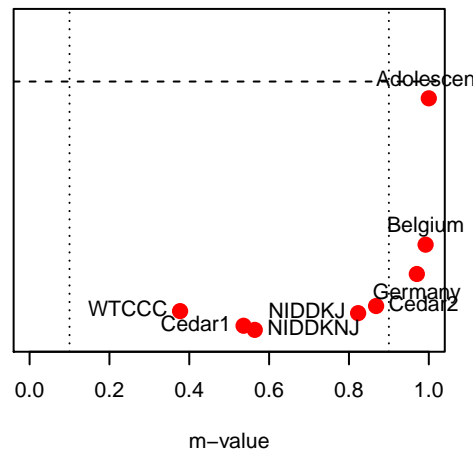

Supplement: Figure S1 — P-M plots of the Crohns disease meta-analysis results of Franke et al. [13]. Six loci showing high heterogeneity are plotted. The names of the studies follow Franke et al. [13]. The dashed horizontal line shows the genome-wide significance threshold. The dotted vertical lines show the prediction regions based on m-value. (PDF) [file pgen.1002555.s001.pdf]

$\alpha=1, \beta=1$

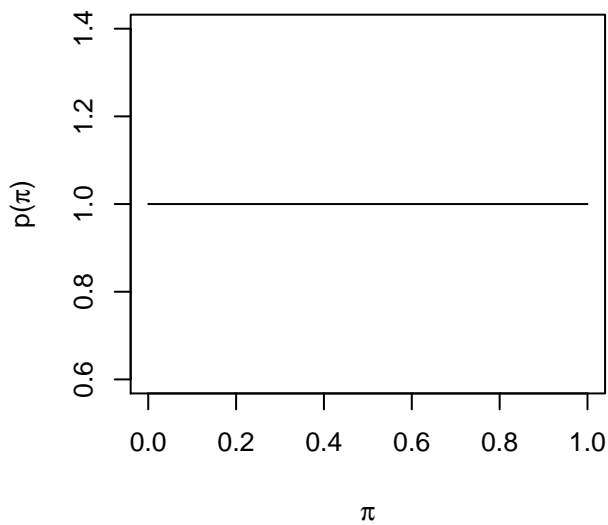

$\alpha=1.2, \beta=1$

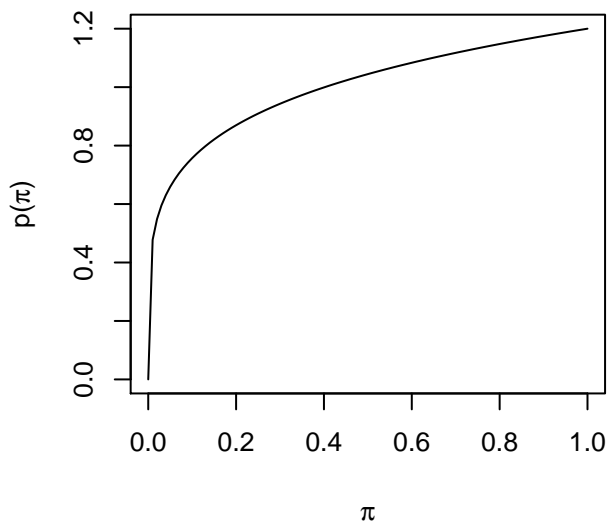

$\alpha=1.5, \beta=1$

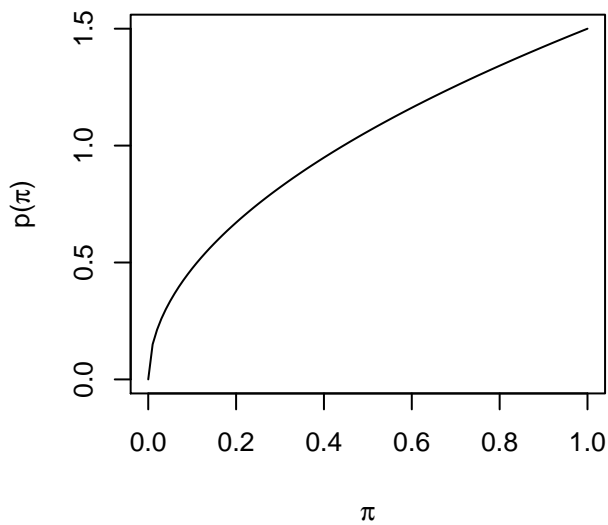

$\alpha=1.5, \beta=1.5$

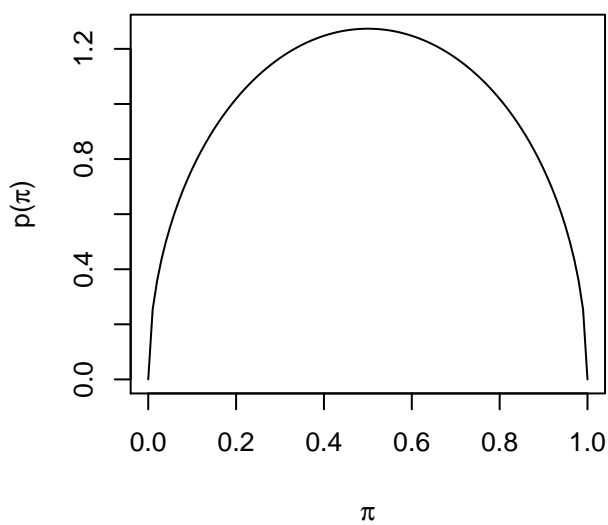

Supplement: Figure S2 — Possible choices for the prior of the probability that the effect exists. We show the uniform distribution prior (), an asymmetric prior preferring the situation that all studies have an effect (), an asymmetric prior preferring the same situation even stronger (), and a bell-shape prior preferring the situation that the studies having an effect and the studies not having an effect are mixed (). (PDF) [file pgen.1002555.s002.pdf]
