# Supplementary material for: Interpreting Meta-Analyses of Genome-Wide Association Studies
Source: PLoS Genet. 2012 Mar 1;8(3):e1002555. doi: 10.1371/journal.pgen.1002555 (PMC3291559; doi:10.1371/journal.pgen.1002555)
Supplement: Supporting Information S1 — This text file includes supporting information for three subjects: (Text S1) Details of the analytical calculation of m-value, (Text S2) P-value estimation using importance sampling for binary effects model, and (Text S3) Efficient m-value approximation for binary effects model. (PDF) [file pgen.1002555.s003.pdf]

Supplementary Information S1: Interpreting Meta-Analyses of Genome-wide Association Studies

Buhm Han[1], Eleazar Eskin[1,2,*]

1 Department of Computer Science, University of California, Los Angeles, Los Angeles, California, United States of America

2 Department of Human Genetics, University of California, Los Angeles, Los Angeles, California, United States of America

∗ Corresponding Author E-mail: eeskin@cs.ucla.edu

Text S1 Details of the analytical calculation of m-value

To analytically work on the integration

$$h(X,t) = \int_{-\infty}^{\infty} \prod_{i \in t_1} N(X_i; \mu, V_i) p(\mu) d\mu \ ,$$

we use the fact that the product of two Gaussian probability density functions results in a single Gaussian [1, 2]. This result can easily be generalized to more than two Gaussians. That is,

$$N(x; \mu_1, V_1) N(x; \mu_2, V_2) ... N(x; \mu_n, V_n) \propto N(x; \mu_{\text{new}}, V_{\text{new}})$$

where

$$\mu_{\text{new}} = \frac{\sum W_i \mu_i}{W_i}$$

$$V_{\text{new}} = \frac{1}{\sum W_i}$$

and $W_i = V_i^{-1}$ is the inverse variance or precision. Also, since a normal distribution is symmetric,

$$N(a; b, V) = N(b; a, V) \ .$$

Using these two facts, given $\mu \sim N(0, \sigma^2)$,

$$\begin{aligned}
h(X,t) &= \int_{-\infty}^{\infty} \prod_{i \in t_1} N(X_i; \mu, V_i) p(\mu) d\mu \\
&= \int_{-\infty}^{\infty} \prod_{i \in t_1} N(\mu; X_i, V_i) p(\mu) d\mu \\
&= \bar{C} \int_{-\infty}^{\infty} N(\mu; \bar{X}, \bar{V}) p(\mu) d\mu \\
&= \bar{C} \int_{-\infty}^{\infty} N(\bar{X}; \mu, \bar{V}) p(\mu) d\mu \\
&= \bar{C} \cdot N(\bar{X}; 0, \bar{V} + \sigma^2)
\end{aligned}$$

where

$$\bar{X} = \frac{\sum_i W_i X_i}{\sum_i W_i}$$

$$\bar{V} = \frac{1}{\sum_i W_i}$$

and $\bar{C}$ is a scaling factor such that

$$\bar{C} = \frac{1}{(\sqrt{2\pi})^{N-1}} \sqrt{\frac{\prod_i W_i}{\sum_i W_i}} \exp\left\{-\frac{1}{2}\left(\sum_i W_i X_i^2 - \frac{(\sum_i W_i X_i)^2}{\sum_i W_i}\right)\right\} .$$

The summations are all with respect to $i \in t_1$.

Text S2 P-value estimation using importance sampling for binary effects model

We suggest an importance sampling procedure for estimating the p-value of the binary effects model. Importance sampling is a statistical technique for reducing the variance of the estimate by sampling from a distribution different from the distribution of interest [3].

In an importance sampling procedure for obtaining p-value, the sampling distribution is chosen so that the sampled statistic can easily exceed the threshold of the observed statistic $\widehat{S}$. In the case of 1-dimensional distribution, this goal is usually achieved by choosing a sampling distribution centered at or around $\widehat{S}$. However, we have a complication that the sampling space is N-dimensional where N is the number of studies in the meta-analysis. Different regions spread over this N-dimensional space can give the same statistic. Therefore, simply changing the center of the original sampling distribution from zero to $\widehat{S}$ will not work, and the importance sampling procedure should effectively traverse all regions. The idea we suggest to achieve this goal is to sample the number of studies having an effect first and sample from the corresponding region, as shown in the following.

First, we sample the number of studies having an effect N_E. We assume a uniform distribution $P(N_E = k) = 1/N$ where $k = 1, ..., N$. Next, we sample N z-scores from the following mixture of normal distributions,

$$f_{N_E}(x) = \frac{N - N_E}{N} \frac{1}{2\pi} e^{-x^2/2} + \frac{N_E}{N} \frac{1}{2\pi} e^{-\left(x - \frac{\widehat{S}}{\sqrt{N_E}}\right)^2/2}$$

where $\widehat{S}$ is the observed binary effects model statistic. The mean value $\frac{\widehat{S}}{\sqrt{N_E}}$ comes from the intuition that if the sample size and the minor allele frequency are the same between studies (V_i is the same between studies) and if the m-values correctly predict the effects in the studies (m_i is one for the studies having effect and zero otherwise), then having N_E z-scores of value $\frac{\widehat{S}}{\sqrt{N_E}}$ and $N - N_E$ z-scores of value zero will give us the exactly same statistic $\widehat{S}$. Therefore, the use of this mean value will lead us to a region that will give a similar statistic to $\widehat{S}$.

If the sample size is different between studies, we use the following sampling distribution instead.

Given N_E, there are $\binom{N}{N_E}$ possible combinations to choose N_E studies. Assume a single combination among them and let M be the set of indices of N_E studies that are chosen to have an effect. Given M, a z-score vector that will exactly produce the statistic $\widehat{S}$ is

$$\frac{\widehat{S}}{\sqrt{\sum_{i\in M} W_i}}\left(\mathbf{I}(1 \in M)\sqrt{W_1}, \mathbf{I}(2 \in M)\sqrt{W_2}, ..., \mathbf{I}(N \in M)\sqrt{W_N}\right) ,$$

where I is an indicator function. Note that in this vector only N_E elements are non-zero. We iterate all $\binom{N}{N_E}$ combinations and calculate the mean β^* and variance V^* of all possible $N_E \cdot \binom{N}{N_E}$ non-zero elements. The new sampling distribution is then

$$f_{N_E}(x) = \frac{N - N_E}{N}\frac{1}{2\pi}e^{-x^2/2} + \frac{N_E}{N}\frac{1}{2\pi}e^{-(x-\beta^*)^2/(2(1+V^*))} ,$$

taking into account the variation caused by unequal sample sizes. If $\binom{N}{N_E}$ is too large for an exact calculation, we randomly sample 1,000 combinations and estimate β^* and V^*.

Now that we defined f_{N_E} given a sampled value N_E, the overall sampling distribution of a vector of z-scores $Z^* = (z_1^*, z_2^*, ..., z_N^*)$ is

$$f_{\text{sample}}(Z^*) = \sum_{k=1}^{N}\left(P(N_E = k)\prod_{i=1}^{N} f_{N_E}(z_i^*)\right) .$$

Given B sampled z-scores $Z_1^*, Z_2^*, ..., Z_B^*$ from this generative model, the p-value is estimated as

$$p = 2\sum_{j=1}^{B}\frac{\mathbf{I}\left(S(Z_j^*) \geq |\widehat{S}|\right) \cdot f_0(Z_j^*)/f_{\text{sample}}(Z_j^*)}{B}$$

where $f_0(Z) = \frac{1}{(2\pi)^{N/2}}e^{-Z^TZ/2}$ is the original null distribution of z-scores and $S(\cdot)$ denotes the binary effects model statistic given the z-scores. The multiplying factor 2 comes from the fact that the null distribution of S is symmetric.

The fact that the null distribution is symmetric can be shown as follows. We informally describe the reasoning rather than giving a formal proof. Given a point Z giving a binary effects model statistic S, consider that we move to a symmetric point about the origin, $-Z$, in the N dimension space. It can be easily shown that the m-values of the studies do not change because the formula (2) (or the formula (S1) if we use approximation) gives the same value. Thus, the statistic corresponding to $-Z$ will simply be $-S$. Therefore, if we let $K_S \in R^N$ be the set of all points giving a statistic S, the region giving the statistic $-S$ will be the symmetric counterpart, $K_{-S} = \{-Z|Z \in K_S\}$. Given the fact that the null distribution of z-scores ($f_0(Z)$) is symmetric about the origin, the probability of S, $f(S) = \int_{K_S} f_0(X)dX$, will be the same as the probability of $-S$, $f(-S) = \int_{K_{-S}} f_0(X)dX$. In other words, the probability density function of S is symmetric under the null.

Text S3 Efficient m-value approximation for binary effects model

Since the binary effects model involves sampling, the standard procedure for estimating m-value is inefficient. We propose an efficient approximation. We first assume a point prior π for the prior probability that the effect exists. Then, the m-value of study i is simply

$$m_i = \frac{\pi N(X_i; \mu, V_i)}{(1-\pi)N(X_i; 0, V_i) + \pi N(X_i; \mu, V_i)}$$

if we knew the true value of μ.

Instead of assuming a distribution prior on μ, we use an empirical approach that estimates μ from the other $N-1$ studies in the meta-analysis. The inverse-variance-weighted estimate is

$$\widehat{X}_i = \frac{\sum_{j \neq i} W_j X_j}{\sum_{j \neq i} W_j} \ .$$

The variance of $\widehat{X}_i$ is given

$$\widehat{V}_i = \frac{1}{\sum_{j \neq i} W_j} \ .$$

Then we set an emprically estimated prior on μ

$$\mu \sim N(\widehat{X}_i, \widehat{V}_i) \ .$$

The approximated m-value is

$$\begin{aligned} m_i^* &= \frac{\pi \int_{-\infty}^{\infty} N(X_i; \mu, V_i) p(\mu) d\mu}{(1-\pi)N(X_i; 0, V_i) + \pi \int_{-\infty}^{\infty} N(X_i; \mu, V_i) p(\mu) d\mu} \\ &= \frac{\pi N(X_i; \widehat{X}_i, V_i + \widehat{V}_i)}{(1-\pi)N(X_i; 0, V_i) + \pi N(X_i; \widehat{X}_i, V_i + \widehat{V}_i)} \end{aligned} \quad \text{(S1)}$$

The intuition under this empirical approach is that by using the other $N-1$ studies, we can obtain the prior on μ which is independent of X_i. In this sense, our approach has similarities to the leave-one-out cross-validation approach usually used in evaluating statistical prediction methods [3].

The advantage of this empirical approach is that the computation is very efficient. The disadvantage is that the information about μ in study i is not utilized since we only use the other $N-1$ studies. In the case that only study i has an effect, as the sample size of the other studies increases, m_i^* asymptotically converges to π instead of 1 since the other studies do not have any clue about μ. For this reason, we use this empirical approach only for the purpose of efficiently computing the binary effects model statistic and p-value. We use $\pi = 0.5$ for all experiments in this paper.

References

1. Penny WD, Roberts SJ (2000) Variational bayes for 1-dimensional mixture models. Techn Report PARG-00-2, Engineering Science, University of Oxford .
2. Smith JO (2011) Spectral Audio Signal Processing, October 2008 Draft. `http://ccrma.stanford.edu/~jos/sasp/`. Online book.
3. Wasserman L (2004) All of statistics. Springer New York.
